# Efficacy and safety of concentration-controlled everolimus with reduced-dose cyclosporine in Japanese *de novo* renal transplant patients: 12-month results

**DOI:** 10.1186/2047-1440-2-14

**Published:** 2013-07-16

**Authors:** Kota Takahashi, Kazuharu Uchida, Norio Yoshimura, Shiro Takahara, Satoshi Teraoka, Rie Teshima, Catherine Cornu-Artis, Eiji Kobayashi

**Affiliations:** 1 Division of Urology, Department of Regenerative and Transplant Medicine, Graduate School of Medical and Dental Sciences, Niigata University, Niigata, 951-8520, Japan; 2 Department of Organ Transplant Surgery, Aichi Medical University, Aichi, 480-1195, Japan; 3 Transplantation and Regenerative Surgery, Graduate School of Medical Science, Kyoto Prefectural University of Medicine, Kyoto, 602-8566, Japan; 4 Department of Advanced Technology for Transplantation, Osaka University Graduate School of Medicine, Osaka, 565-0871, Japan; 5 Department of Transplant Surgery, International University of Health and Welfare Atami Hospital, Shizuoka, 413-0012, Japan; 6 Novartis Pharma K.K, Tokyo, 106-8618, Japan; 7 Novartis Pharma AG, Basel, CH-4002, Switzerland; 8 Division of Development of Advanced Treatment, Center for Development of Advanced Medical Technology, Jichi Medical University, Tochigi, 329-0498, Japan

**Keywords:** Everolimus, Cyclosporine, Renal transplantation, Renal function, Therapeutic drug monitoring

## Abstract

**Background:**

No study to date has evaluated the efficacy and safety of everolimus with reduced-exposure cyclosporine in Japanese *de-novo* renal transplant (RTx) patients.

**Methods:**

This 12-month, multicenter, open-label study randomized (1:1) 122 Japanese *de-novo* RTx patients to either an everolimus regimen (1.5 mg/day starting dose (target trough: 3 to 8 ng/ml) + reduced-dose cyclosporine) or a mycophenolate mofetil (MMF) regimen (2 g/day + standard dose cyclosporine). All patients received basiliximab and corticosteroids. Key endpoints at month 12 were composite efficacy failure (treated biopsy-proven acute rejection, graft loss, death, or loss to follow-up) and renal function (estimated glomerular filtration rate; Modification of Diet in Renal Disease-4).

**Results:**

Clear cyclosporine exposure reduction was achieved in the everolimus group throughout the study (52% reduction at month 12). Month 12 efficacy failure rates showed everolimus 1.5 mg to be non-inferior to MMF (11.5% vs. 11.5%). The median estimated glomerular filtration rate at month 12 was 58.00 ml/minute/1.73 m^2^ in the everolimus group versus 55.25 ml/minute/1.73 m^2^ in the MMF group (*P* = 0.063). Overall, the incidence of adverse events was comparable between the groups with some differences in line with the known safety profile of the treatments. The everolimus group had a higher incidence of wound healing events and edema, whereas a higher rate of cytomegalovirus infections was reported in the MMF group.

**Conclusions:**

This study confirmed the efficacy of everolimus 1.5 mg/day (target trough: 3 to 8 ng/ml) in Japanese RTx patients for preventing acute rejection, while allowing for substantial cyclosporine sparing. Renal function and safety findings were comparable with previous reports from other RTx populations.

**Trial registration:**

ClinicalTrials.gov number: NCT00658320

## Background

In Japan, the standard immunosuppressive therapy for renal transplant (RTx) patients comprises a quadruple regimen of basiliximab induction with a calcineurin inhibitor (CNI; cyclosporine A (cyclosporine) or tacrolimus), mycophenolate mofetil (MMF) and corticosteroids [1]. This CNI-based regimen remains the mainstay of immunosuppression following kidney transplantation worldwide [2], but improvements in long-term graft survival are restricted by the chronic nephrotoxicity associated with CNI therapy [3,4]. Intense efforts are being made to develop immunosuppressive strategies that permit early CNI minimization or elimination, potentially leading to a reduction in CNI-related nephrotoxicity and other adverse events (AEs) without compromising acute rejection rates.

Everolimus, a mammalian target of rapamycin (mTOR) inhibitor with potent immunosuppressive and antiproliferative effects [5], has shown good efficacy and tolerability when used in combination with a CNI in *de novo* kidney transplant recipients [6-14]. However, coadministration of everolimus with standard-exposure CNI therapy adversely affects renal function due to potentiation of CNI-related nephrotoxicity [7,9,15]. A number of studies have therefore assessed a variety of everolimus-based, CNI-sparing protocols in order to identify the optimal balance between preventing rejection and preserving graft function [10-14]. Results from large, randomized controlled trials have demonstrated the effectiveness of reduced-exposure cyclosporine with an initial everolimus dose of 1.5 mg/day, subsequently adjusted to target an everolimus trough concentration (C0) of 3 to 8 ng/ml [6,12-14]. The most recent of these trials (A2309) confirmed that this regimen was non-inferior in terms of the primary efficacy endpoint to standard-exposure cyclosporine with mycophenolic acid based on a total of 277 patients in each group [14].

These trials, however, were largely conducted in non-Asian patients (87 to 92%). Moreover, a high proportion of grafts were from deceased-donor recipients (45 to 100%), whereas in Japan virtually all solid organ transplants are undertaken with living donors [6,12-14]. A single study has demonstrated that the pharmacokinetics of everolimus are similar in Japanese or non-Japanese volunteers [16], as recommended by the Pharmaceuticals and Medical Devices Agency of Japan [17]. However, clinical trials of a reduced CNI regimen with an everolimus target exposure of 3 to 8 ng/ml are lacking in Japanese or other Asian populations.

The current randomized, multicenter, 12-month study compared the efficacy and safety of *de novo* everolimus with reduced-exposure cyclosporine to MMF with standard-dose cyclosporine in Japanese RTx patients.

## Methods

### Study design

This was a 12-month, multicenter, randomized, open-label study in Japanese adult *de novo* RTx patients. The study was conducted from February 2008 to August 2010. Following eligibility screening, patients were stratified by donor type (deceased donor or living donor) and randomized (1:1) when the graft function was confirmed just after transplantation into either the everolimus group (everolimus 1.5 mg (targeted C0: 3 to 8 ng/ml) + reduced-dose cyclosporine) or the MMF group (MMF 2 g/day + standard-dose cyclosporine) All patients received basiliximab induction therapy + corticosteroids.

The randomization list was produced by an independent clinical research organization using a validated system that automated the random assignment of treatment arms to randomization numbers.

The independent ethics committee at each center approved this study and written informed consent was obtained from each patient before enrollment. The study was conducted and monitored according to Good Clinical Practice guidelines.

### Patients

Patients aged 18 to 65 years undergoing primary kidney transplantation were eligible. Key exclusion criteria included no evidence of graft function within 24 hours of transplantation, patients of kidneys with a cold ischemia time >24 hours; donor age >65 years; patients of multiorgan, ABO-incompatible, positive T-cell cross-match or HLA identical living-related-donor transplants; or most recent anti-HLA class I panel-reactive antibodies >20% by complement-dependent cytotoxicity-based assay or >50% by flow cytometry or ELISA.

### Immunosuppression and other concomitant medications

The initial dose of the study medication was given within 24 hours (if difficult due to the patient’s condition, then within 36 hours) post transplantation. From day 5 onwards, cyclosporine dose adjustments were made based on C0 (determined by local laboratory). Target cyclosporine C0 concentrations in the everolimus 1.5 mg group started with 100 to 200 ng/ml and were lowered to 75 to 150 ng/ml starting at month 2, then 50 to 100 ng/ml starting at month 4, and 25 to 50 ng/ml from month 6 onwards. In the MMF group, patients started with a cyclosporine C0 target concentration of 200 to 300 ng/ml, which was lowered to 100 to 250 ng/ml starting at month 2 with this target range to be maintained for the remainder of the study. Everolimus doses were adjusted from day 5 onwards to maintain a C0 targeted at 3 to 8 ng/ml (measured by the central laboratory). Therapeutic drug monitoring was mandatory throughout the duration of the study. All patients received basiliximab (20 mg) within 2 hours prior to transplantation and at day 4, or according to local practice. Corticosteroids were administered according to local practice, at a minimum dose of 5 mg/day for 12 months. Cytomegalovirus (CMV) prophylaxis (including pre-emptive therapy) was mandatory for all cases in which the donor tested positive and the recipient tested negative for CMV. The duration of prophylaxis was not defined in the protocol. All cases other than CMV-positive donors and CMV-negative recipients were treated according to local practice. The drug and dose of the therapy were not defined in the protocol and were according to local practice of the study site. CMV prophylaxis was also recommended following any antibody treatment of acute rejection episodes.

### Study endpoints

#### Efficacy

The primary endpoint was efficacy failure, defined as the composite of treated biopsy-proven acute rejection (BPAR), graft loss, death or loss to follow-up (LTFU) at month 12; LTFU was defined as a patient who did not experience treated BPAR, graft loss or death and whose last day of contact was prior to the month 12 visit. The main secondary endpoint was the composite of graft loss, death or LTFU at month 12. In all suspected rejection episodes, a graft core biopsy (read by the local and central pathologists according to the updated Banff’97 criteria [18]) was performed within 48 hours. The treated BPAR endpoint was assessed using the central pathologists’ reading.

#### Safety

The main safety endpoint was renal function at month 12. Renal function was determined by estimated glomerular filtration rate (eGFR) using the Modification of Diet in Renal Disease formula [19]. Other safety assessments included reported AEs and serious AEs as well as clinical laboratory measurements and vital signs.

### Statistical analyses

The primary efficacy analyses were conducted on the intent-to-treat population (all patients randomized). The non-inferiority test was based on confidence intervals (CIs) constructed using the Z-test statistic. One-sided 95% CIs and two-sided 90% CIs for the difference in primary efficacy failure rates at 12 months between the everolimus and MMF arms were computed. Everolimus was considered non-inferior to MMF if the upper limit of the 95% CI was less than 13%. Kaplan–Meier survival analyses were performed on the rates of composite efficacy failure and its components. For each of the secondary efficacy endpoints, simple event rate estimates (proportion of events) were compared for the everolimus group with the MMF group using Z-statistics based on one-sided 95% CIs for differences in event rates. The eGFR at 12 months was summarized using descriptive statistics. The Wilcoxon rank-sum test was used to compare the two groups. Except for the renal function analyses, safety analyses were performed on the safety population (patients who received at least one dose of study drug and had a post baseline safety assessment).

### Sample size calculation

The efficacy failure rates at month 12 for the everolimus and MMF groups were assumed to be 19% and 20%, respectively. Owing to the limited number of RTx patients available, a maximum of 120 patients (60 patients/arm) were expected to be enrolled. A sample size of 60 patients per arm had 61% power to show everolimus was statistically non-inferior to MMF at one-sided 0.05 levels and non-inferiority margin 13%.

## Results

### Patient disposition

**Figure 1:**
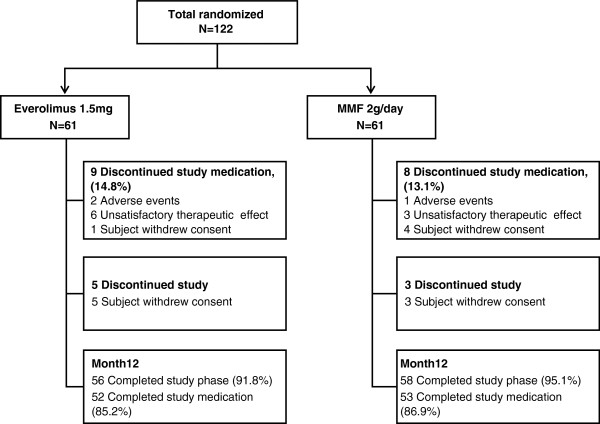
**Patient disposition.** MMF, mycophenolate mofetil.

The study population included a total of 122 patients, randomized 1:1 to the everolimus (*n* = 61) and MMF (*n* = 61) groups (intent-to-treat population; safety population). More than 90% of patients completed the study in both treatment groups and more than 85% of patients completed the 12-month period on study medication. A total of eight patients discontinued the study at month 12 and all of the study discontinuations were due to withdrawal of consent (Figure 1). Overall, demographic and baseline characteristics were comparable between the groups and are presented in Table 1. The mean age was 42.5 years for the everolimus group and 38.6 years for the MMF group. The majority of patients were male (75.4% of everolimus patients and 60.7% of MMF patients). Donor characteristics were generally similar for both groups. The mean age of donors was 52.3 years for the everolimus group and 55.3 years for the MMF group. Except for one deceased donor each in both the groups, all donors were living and the majority of the donors were living related (59.0% in the everolimus group and 70.5% in the MMF group).

**Table 1:** Summary of patient demographics and kidney transplantation background by treatment group (intent-to-treat population)

	**Everolimus 1.5 mg (*n *= 61)**	**MMF 2 g (*n *= 61)**
**Recipient characteristics**		
Age (years)		
Mean ± standard deviation	42.5 ± 14.13	38.6 ± 11.36
Median (range)	42.0 (18 to 65)	36.0 (20 to 64)
Gender, *n* (%)		
Male	46 (75.4)	37 (60.7)
Female	15 (24.6)	24 (39.3)
Body mass index (kg/m^2^)		
Mean ± standard deviation	22.46 ± 4.03	21.79 ± 2.78
Median (range)	21.97 (15.5 to 37.5)	21.09 (16.0 to 27.6)
Primary disease leading to transplantation, *n* (%)		
Glomerulonephritis/glomerular disease	16 (26.2)	9 (14.8)
Polycystic disease	3 (4.9)	3 (4.9)
Hypertension/nephrosclerosis	5 (8.2)	2 (3.3)
Diabetes mellitus	3 (4.9)	5 (8.2)
Interstitial nephritis	2 (3.3)	0 (0.0)
Obstructive disorder/reflux	6 (9.8)	3 (4.9)
IgA nephropathy	11 (18.0)	16 (26.2)
Unknown	8 (13.1)	16 (26.2)
Other	7 (11.5)	7 (11.5)
Current dialysis		
None	12 (19.7)	8 (13.1)
Hemodialysis	42 (68.9)	48 (78.7)
Peritoneal dialysis	7 (11.5)	5 (8.2)
HLA mismatches		
1	7 (11.5)	2 (3.3)
2	9 (14.8)	16 (26.2)
3	25 (41.0)	24 (39.3)
<3	16 (26.2)	18 (29.5)
≥3	45 (73.8)	43 (70.5)
**Donor characteristics**		
Mean ± standard deviation age (years)	52.3 ± 8.99	55.2 ± 8.23
Deceased heart beating, *n* (%)	1 (1.6)	0 (0.0)
Deceased nonheart beating, *n* (%)	0 (0.0)	1 (1.6)
Living related, *n* (%)	36 (59.0)	43 (70.5)
Living unrelated, *n* (%)	24 (39.3)	17 (27.9)

*HLA*, human leukocyte antigen; *IgA*, immunoglobulin A; *MMF*, mycophenolate mofetil.

### Immunosuppressant dose and exposure

**Figure 2:**
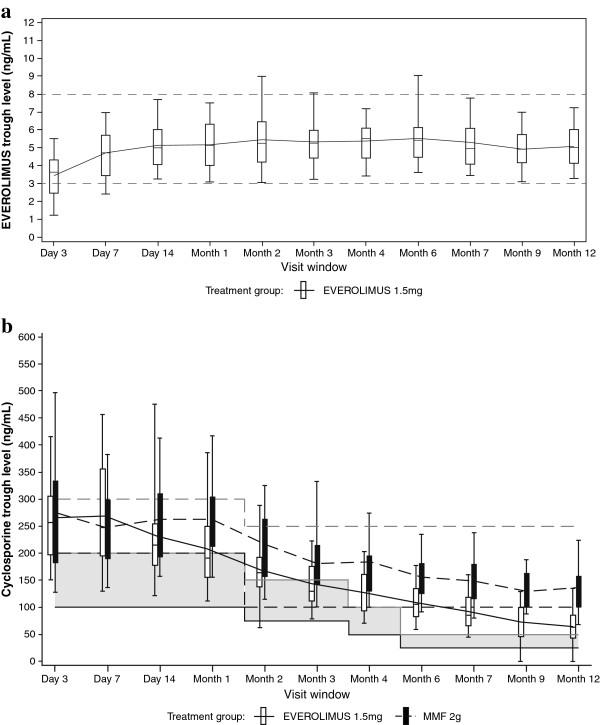
**Mean everolimus and cyclosporine trough levels over time.** (**a**) Mean everolimus trough levels over time for the everolimus 1.5 mg group (safety population). At each visit the mean is shown and these are joined with horizontal lines. Whiskers are the 5th and 95th percentiles. Target levels are displayed. (**b**) Mean cyclosporine trough levels for all treatment groups over time (safety population). At each visit the mean is shown and these are joined with horizontal lines. Whiskers are the 5th and 95th percentiles. The target ranges are also displayed with solid lines (lower limit) and broken lines (upper limit) for comparison with the values seen. MMF, mycophenolate mofetil.

The majority of everolimus patients (>85% from day 7 onwards) were maintained within the targeted everolimus exposure, with the mean everolimus C0 ranging from 3.4 to 5.5 ng/ml (Figure 2a). Although a higher proportion of everolimus patients were above the cyclosporine target range versus the MMF group, a clear separation of cyclosporine exposure was achieved between the everolimus group and the MMF group throughout the study period with a 52% lower mean and median exposure in the everolimus group at month 12 (median: 63.0 ng/ml and 130.5 ng/ml, respectively) (Figure 2b). The mean MMF doses were decreased up to month 3 due to the adverse events, and subsequently were kept constant throughout the study. The mean ± standard deviation dose of MMF at month 12 was 1.24 ± 0.530 g/day. Mean everolimus and cyclosporine trough levels and average daily doses of everolimus and MMF from day 3 to month 12 are shown in Figure 2a,b and Table 2.

**Table 2:** Average daily doses of everolimus (mg/day) and mycophenolate mofetil (g/day) by visit window (safety population)

	**Everolimus 1.5 mg (*n *= 61)**		**MMF (*n *= 61)**	
**Visit window**	***n***	**Mean ± SD**	***n***	**Mean ± SD**
Day 3	61	1.49 (0.0.048)	61	1.95 (0.194)
Day 7	60	1.70 (0.447)	61	1.88 (0.297)
Month 1	57	1.72 (0.539)	60	1.70 (0.475)
Month 3	55	1.70 (0.628)	58	1.33 (0.606)
Month 4	55	1.68 (0.611)	56	1.22 (0.595)
Month 6	55	1.65 (0.602)	55	1.28 (0.583)
Month 7	55	1.61 (0.610)	54	1.28 (0.570)
Month 9	54	1.68 (0.656)	54	1.25 (0.530)
Month 12	53	1.68 (0.705)	53	1.24 (0.530)

*MMF*, mycophenolate mofetil; *SD*, standard deviation.

### Efficacy

#### Primary efficacy endpoint

Composite efficacy failure event rates at month 12 were identical in both groups (everolimus, 11.5% and MMF, 11.5%; Table 3). This treatment difference of 0.0% with the upper limit of the 95% CI at 9.5% was lower than the predefined non-inferiority margin of 13% confirming non-inferiority of the everolimus group to the MMF group. The Kaplan–Meier plot of the proportion of patients free from composite efficacy failure over the 12-month period confirmed similar efficacy for the everolimus and MMF groups (see Additional file 1). There were four patients in the everolimus group and two patients in the MMF group who withdrew informed consent to participate in the study and for whom no further information could be collected. These patients were accounted for as lost to follow-up. There were two patients, one in each group, who developed treated BPAR before discontinuation of the study due to withdrawal of consent, and hence they were not included in the loss to follow-up category.

**Table 3:** Summary of efficacy parameters by treatment (intent-to-treat population)

	**Everolimus 1.5 mg (*n *= 61)**	**MMF 2 g (*n *= 61)**	**Comparison of everolimus vs. MMF**
**Efficacy endpoints**			
Primary composite endpoint (at 12 months)^a^	7 (11.5)	7 (11.5)	Difference in rates 0.0% (9.5%), (−9.49, 9.49) *P* = 0.012^d^
Treated BPAR	3 (4.9)	5 (8.2)	
Graft loss	0	0	
Death	0	0	
Loss to follow-up^b^	4 (6.6)	2 (3.3)	
**Secondary efficacy endpoints**			
Patients with treated BPAR by Banff grade			
IA	2 (3.3)	2 (3.3)	
IB	0	1 (1.6)	
IIA	1 (1.6)	2 (3.3)	
Graft loss or death (month 12)	0	0	
Graft loss, death or loss to follow-up^c^ (month 12)	5 (8.2)	3 (4.9)	3.3% (10.6%), *P* = 0.015^d^

Data presented as *n* (%). ^a^Composite of treated BPAR, graft loss, death, or loss to follow-up. For the individual components of the composite endpoint, patients are counted for the first event to occur. ^b^A loss to follow-up in the primary endpoint is a patient who did not experience treated BPAR, graft loss, or death and whose last day of contact was prior to day 316 (that is, prior to the month 12 visit window). ^c^A loss to follow-up in the secondary endpoint is a patient who did not experience graft loss or death and whose last day of contact was prior to day 316 (that is, prior to the month 12 visit window). Note that for patients meeting the composite efficacy endpoint, patients are recorded by the individual. ^d^Z-test for everolimus – MMF ≥0.13 (non-inferiority test, *P* value for non-inferiority test is for a one-sided test and should be compared with the 0.05 significance level. BPAR, biopsy-proven acute rejection; MMF, mycophenolate mofetil.

### Secondary efficacy endpoints

The main secondary efficacy endpoint (combined rate of death, graft loss and LTFU) at month 12 was statistically non-inferior for everolimus (8.2%) versus MMF (4.9%). All of the events were due to LTFU with no cases of graft loss or death (Table 3). Treated BPAR (based on central biopsy readings) occurred in three (4.9%) everolimus patients versus five (8.2%) MMF patients. The majority of the treated BPARs were of Banff type 1A in the everolimus group (Table 3). These results were confirmed based on local biopsy results with treated BPAR occurring in four everolimus patients versus eight MMF patients, with the majority of treated BPARs of type IA or IB.

### Safety

#### Renal function

Median eGFR at month 12 was 58.00 ml/minute/1.73 m^2^ with everolimus versus 55.25 ml/minute/1.73 m^2^ with MMF (*P* = 0.063). For both treatment groups, the mean and median eGFR gradually increased at a similar rate during the first month after transplantation. The eGFR levels were higher for the everolimus group through the study but the treatment comparisons did not show any statistically significant differences between the groups at any time point (Table 4; see also Additional file 2). The chronic kidney disease category was used as a guide to evaluate the renal function. The proportion of patients with month 12 eGFR ≥60 ml/minute/1.73 m^2^ was higher with everolimus (46.4%) compared with MMF (32.8%), but it was not statistically significant (*P* = 0.152) (Table 4).

**Table 4:** Renal function over 12 months (intent-to-treat population)

**Visit window**	**Everolimus 1.5 mg (*n *= 61)**				**MMF 2 g (*n *= 61)**		
	***n***	**Mean (SD)**	**Median (range)**	***P *value**^**a **^**vs. MMF**	***n***	**Mean (SD)**	**Median (range)**
**eGFR (MDRD) (ml/minute/1.73 m**^**2**^**)**							
Baseline	61	12.17 (6.23)	10.70 (4.7 to 41.1)	0.420	61	14.00 (8.37)	11.00 (2.7 to 41.1)
Month 1	56	63.10 (25.441)	58.40 (18.5 to 123.3)	0.685	60	60.53 (19.339)	57.25 (23.5 to 14.7)
Month 12	56	62.09 (18.993)	58.00 (17.8 to 123.3)	0.063	58	56.34 (15.227)	55.25 (26.1 to 111.8)
	**<30**	**≥30 to <60**	**≥60**	***P *value vs. MMF**	**<30**	**≥30 to <60**	**≥60**
**Incidence rates of patients within renal function (eGFR MDRD) categories ( *n */month, %)**							
Month 1	5/56 (8.9)	26/56 (46.4)	25/56 (44.6)	0.541	1/60 (1.7)	31/60 (51.7)	28/60 (46.7)
Month 12	1/56 (1.8)	29/56 (51.8)	26/56 (46.4)	0.152	1/58 (1.7)	38/58 (65.5)	19/58 (32.8)

^a^Wilcoxon rank-sum test comparing everolimus and MMF values. *eGFR*, estimated glomerular filtration rate; *MDRD*, Modification of Diet in Renal Disease; *MMF*, mycophenolate mofetil; *SD*, standard deviation.

#### Adverse events and laboratory parameters

The overall incidence of AEs was comparable between the treatment groups (Table 5). The proportion of patients reporting any serious AEs was approximately 10% higher for the MMF group (54.1%) versus the everolimus group (44.3%) (risk ratio (RR) = 0.82 (95% CI = 0.568, 1.178)). A higher proportion of the MMF patients (26.2%) versus the everolimus (19.7%) patients (RR =0.75 (95% CI = 0.388, 1.450)) experienced serious infections, particularly serious CMV infections (18.0% vs. 1.6%, respectively, RR = 0.09 (0.012, 0.683)), gastroenteritis (6.6% vs. 3.3%, respectively), and herpes zoster infections (3.3% vs. 0%, respectively).

**Table 5:** Summary of adverse events over 12 months of treatment (safety population)

	**Everolimus 1.5 mg (*n *= 61)**	**MMF 2 g (*n *= 61)**	**Risk ratio (95% CI)**
Any adverse event	61 (100)	61 (100)	–
Serious adverse events	27 (44.3)	33 (54.1)	0.82 (0.568, 1.178)
Severe adverse events	7 (11.5)	8 (13.1)	0.88 (0.338, 2.263)
Adverse events leading to study drug discontinuation^a^	3 (4.9%)	1 (1.6%)	3.00 (0.321, 28.044)
Adverse events leading to study drug dose adjustment/interruption	15 (24.6)	52 (85.2)	0.29 (0.184, 0.453)
**Most frequently reported adverse events and infections (≥20% of patients in any treatment group)**^**b**^			
Hyperlipidemia	28 (45.9)	19 (31.1)	1.47 (0.928, 2.339)
Nasopharyngitis	21 (34.4)	26 (42.6)	0.81 (0.514, 1.270)
Constipation	19 (31.1)	27 (44.3)	0.70 (0.441, 1.123)
Hypertension	19 (31.1)	18 (29.5)	1.06 (0.616, 1.808)
Insomnia	17 (27.9)	9 (14.8)	1.89 (0.914, 3.903)
Acne	15 (24.6)	22 (36.1)	0.68 (0.393, 1.184)
Headache	13 (21.3)	9 (14.8)	1.44 (0.667, 3.127)
Toxic nephropathy	13 (21.3)	6 (9.8)	2.17 (0.881, 5.329)
Blood alkaline phosphatase increased	13 (21.3)	7 (11.5)	1.86 (0.796, 4.334)
Pyrexia	13 (21.3)	12 (19.7)	1.08 (0.538, 2.181)
Iron deficiency anemia	12 (19.7)	13 (21.3)	0.92 (0.458, 1.858)
Diarrhea	11 (18.0)	15 (24.6)	0.73 (0.367, 1.466)
Increased blood creatinine	11 (18.0)	14 (23.0)	0.79 (0.388, 1.591)
Hyperuricemia	7 (11.5)	13 (21.3)	0.54 (0.231, 1.257)
Cytomegalovirus test positive	4 (6.6)	19 (31.1)	0.21 (0.076, 0.583)
Cytomegalovirus infection	3 (4.9)	21 (34.4)	0.14 (0.045, 0.454)
**Other adverse events of interest**			
Cyclosporine-associated adverse events			
Gingival hypertrophy	0 (0.0)	2 (3.3%)	–
Gingival injury	0 (0.0)	1 (1.6%)	–
Gingivitis	0 (0.0)	1 (1.6%)	–
Tremor	4 (6.6%)	1 (1.6%)	4.00 (0.460, 34.767)
Hirsutism	1 (1.6%)	4 (6.6%)	0.25 (0.029, 2.173)
Hypertrichosis	2 (3.3%)	3 (4.9%)	0.67 (0.115, 3.850)
Everolimus-associated adverse events			
Wound-healing event^c^	24 (39.3)	7 (11.5)	3.43 (1.598, 7.357)
New-onset diabetes^c^	7 (11.5)	3 (4.9)	2.33 (0.633, 8.606)
Edema events^c^	20 (32.8)	8 (13.1)	2.50 (1.194, 5.235)
Stomatitis events^c^	14 (23.0)	10 (16.4)	1.40 (0.675, 2.904)
Blood luteinizing hormone increased	9 (14.8)	0 (0.0)	–
Blood follicle stimulating hormone increased	8 (13.1)	1 (1.6)	8.00 (1.032, 62.040)
Proteinuria	8 (13.1)	5 (8.2)	1.60 (0.555, 4.616)
Investigator-reported severity			
Mild	6 (9.8)	3 (4.9)	2.00 (0.524, 7.636)
Moderate	2 (3.3)	1 (1.6)	2.00 (0.186, 21.482)
Severe	0 (0.0)	1 (1.6)	–

Data are presented as *n* (%). ^a^For patients with adverse events leading to discontinuation of study medication (recorded on the AE/Infection CRF page), the primary discontinuation reason (recorded on the End of Treatment CRF page) is not necessarily ‘AE(s)’; rather, it may be ‘abnormal laboratory result(s)’ or ‘unsatisfactory therapeutic effect’. ^b^By preferred term. ^c^Events were identified from a predefined list of adverse event preferred terms. *MMF*, mycophenolate mofetil.

The most common AEs were nasopharyngitis, hyperlipidemia, constipation, acne and hypertension, and the majority of AEs (>85% in either group) were mild or moderate in severity. AEs/infections leading to discontinuation of the study drug occurred in 4.9% of the patients in the everolimus group (pyrexia, diffuse large B-cell lymphoma and membranous glomerulonephritis) versus 1.6% in the MMF group (electrolyte imbalance and hirsutism) (Table 5). A higher proportion of the MMF patients (85.2%) versus the everolimus patients (24.6%) had AEs requiring study drug dose adjustment/interruption (Table 5). This was mostly due to infections (52.5% in MMF group vs. 13.1% in everolimus group). The incidence of any infection was higher with MMF (93.4%) versus everolimus (82.0%). Viral infections were more frequent in the MMF group (80.3%) compared with the everolimus group (27.9%) (RR = 0.35 (95% CI = 0.227, 0.529)), predominantly due to the higher rate of CMV (68.9% vs. 14.8%) (see Additional file 3). Only one patient in the everolimus group (CMV-positive donor/CMV-negative recipient) and six patients in the MMF group (three CMV-positive donor/CMV-negative recipient and three CMV-positive donor/CMV-positive recipient) received CMV prophylaxis (see Additional file 4).

The incidence of toxic nephropathy reported as cyclosporine nephrotoxicity was numerically higher in the everolimus group (21.3%) than in the MMF group (9.8%) (RR = 2.17 (95% CI = 0.881, 5.329)) (Table 5). Malignancies were reported for two (3.3%) everolimus patients (one patient with thyroid cancer and one patient with B-cell lymphoma), whereas no malignancies were reported in MMF patients. Wound healing events were reported for 24 (39.3%) everolimus patients and seven (11.5%) MMF patients (RR = 3.43 (95% CI = 1.598, 7.357)). Wound events were reported as serious AEs in three (4.9%) everolimus patients and one (1.6%) MMF patient. Most of the wound healing events reported were mild (4.9% and 1.6%) to moderate (34.4% and 8.2%), with 0% and 1.6% of events classified as severe in the everolimus and the MMF groups, respectively. The most common wound healing AE was lymphocele, which was reported for seven (11.5%) everolimus patients and two (3.3%) MMF patients. Impaired healing was reported as an AE for six (9.8%) everolimus patients and one (1.6%) MMF patient. Edema occurred in 20 (32.8%) everolimus and eight MMF patients (13.8%) (RR = 2.50 (95% CI = 1.194, 5.235)). Proteinuria was reported as an AE in eight (13.1%) everolimus patients and five (8.2%) MMF patients (RR = 1.60 (95% CI = 0.555, 4.616)) (Table 5). The urinary protein:creatinine ratio in the everolimus group was slightly higher than in the MMF group throughout the study (median at month 12 was 135.0 mg/g and 65.0 mg/g, respectively). AEs generally associated with cyclosporine were more frequently reported in the MMF group versus the everolimus group (Table 5).

The mean and median systolic blood pressure and diastolic blood pressure decreased from baseline for both treatment groups (Table 6). Low neutrophil counts (≤1,000/mm^3^) were observed for two (3.3%) MMF patients. Hyperlipidemia was reported in 28 (45.9%) everolimus patients and 19 (31.1%) MMF patients, and hypercholesterolemia was reported for seven (11.5%) everolimus patients and six (9.8%) MMF patients. High triglyceride levels (≥750 mg/dl) were reported for one (1.6%) everolimus patient and high total cholesterol levels (>350 mg/dl) for two (3.3%) everolimus patients and one (1.6%) MMF patient (Table 6).

**Table 6:** Vital signs, hematological and biochemical abnormalities over 12 months of treatment (safety population)

	**Everolimus 1.5 mg (*n *= 61)**	**MMF 2 g (*n *= 61)**
SBP (mmHg)		
≤90 mmHg or <75 mmHg^a^	1 (1.6)	0 (0.0)
≥180 mmHg or >200 mmHg^b^	9 (14.8)	6 (9.8)
DBP (mmHg)		
≤50 mmHg or <40 mmHg	4 (6.6)	0 (0.0)
≥105 mmHg or >115 mmHg	14 (23.0)	16 (26.2)
Hematology		
Platelets, low: <50 k/mm^3^	0 (0.0)	1 (1.6)
Eosinophils, high: ≥12%	1 (1.6)	1 (1.6)
Hemoglobin, low: <7 g/dl	5 (8.2)	5 (8.2)
Lymphocytes, low: ≤1,000/mm^3^	48 (78.7)	56 (91.8)
Leukocytes		
Low: ≤2.0 k/mm^3^	0 (0.0)	1/61(1.6)
High: ≥16 k/mm^3^	32 (52.5)	20 (32.8)
Neutrophils, low: ≤1,000/mm^3^	0 (0.0)	2 (3.3)
Lipids		
Total cholesterol, high: ≥350 mg/dl	2 (3.3)	1 (1.6)
Triglycerides, high: ≥750 mg/dl	1 (1.6)	0 (0.0)
Cholesterol (total)/HDL ratio		
High: ≥5 and ≤7	24 (39.3)	17 (27.9)
Very high: >7	5 (8.2)	3 (4.9)
Lipid modifying agents	42 (68.9)	24 (39.3)
Number of patients with normalized cholesterol values after statin treatment (*n*/month)	16/18 (88.9)	6/7 (85.7)
Number of patients with normalized triglyceride values after statin treatment	9/12 (75)	3/4 (75)

Data presented as *n* (%). ^a^Decrease from baseline ≥30 mmHg. ^b^Increase from baseline ≥30 mmHg. *DBP*, diastolic blood pressure; *HDL*, high-density lipoprotein; *SBP*, systolic blood pressure.

## Discussion

Results of this randomized study in Japanese *de novo* kidney transplant patients indicate that everolimus with reduced-exposure cyclosporine provides similar efficacy, renal function and safety to MMF with standard-exposure cyclosporine over the first 12 months post transplant. These outcomes were achieved with ~52% lower cyclosporine A trough concentration in the everolimus treatment group versus the standard therapy arm. The findings from this trial are comparable with those reported in a large, predominantly Caucasian population in the recent A2309 study [12].

There were no graft losses or deaths in either treatment group, and rates of treated BPAR at 12 months were notably low in both treatment arms (everolimus ~5%, MMF ~8%). The low incidence of rejection partly reflects the almost universal use of living donors in our cohort, but slightly higher cyclosporine exposure than in the recent A2309 study [12] may also have contributed. This was balanced by a somewhat lower mean everolimus trough concentration in the current trial. Recipient demographics were broadly similar in the two studies, although donor age was slightly older in the current trial.

The key renal endpoint, eGFR (Modification of Diet in Renal Disease) at month 12, did not differ significantly between the two treatment groups (*P* = 0.063) but was numerically higher in the everolimus cohort throughout the trial. As might be expected in our living-donor population, the mean eGFR was slightly higher than in the larger A2309 study, but the pattern of difference between treatment groups was comparable. The A2309 trial also showed the mean eGFR to be numerically higher in the everolimus-treated patients at all time points, but in that larger study population the between-group difference became significant at months 1, 6, 7 and 9. In both trials, the proportion of patients with CKD stage ≥4 (that is, eGFR ≥60 ml/minute/1.73 m^2^) at month 12 was higher in the everolimus group versus the mycophenolic acid cohort, an encouraging finding since renal function at 12 months post RTx is recognized as predictive of long-term renal function [20]. No everolimus-treated patient was reported to have severe proteinuria in our population. The incidence of toxic nephropathy was higher with everolimus versus MMF, a difference that arose during the first 14 days after transplantation. In that 2-week period, mean cyclosporine trough levels in the everolimus group were no different from those in the MMF group. Since everolimus is known to potentiate cyclosporine-related nephrotoxicity when cyclosporine exposure is high [7,9,15], the acute nephrotoxicity that was observed may probably have been largely caused by high cyclosporine exposure. Previously it has been reported that CNI-associated acute nephrotoxicity early after transplant can be resolved with dose reduction or interruption [21,22]. In this study with subsequent reductions in cyclosporine exposure, the eGFR for the everolimus group was higher than for the MMF group at month 12 post transplantation, and no difference in the rate of chronic nephrotoxicity was reported between the two groups, highlighting the importance of prompt and adequate CNI reduction in the presence of everolimus.

The overall safety profile of everolimus was similar to that seen in previous studies and no AEs were identified that appeared to be specific to Japanese patients. Hyperlipidemia, insomnia, increased alkaline phosphatase, increased luteinizing hormone and follicle stimulating hormone, wound healing events and edema were more frequent with everolimus, while the incidences of CMV infection, nasopharyngitis, constipation and acne were all higher with MMF. The incidence of serious AEs was approximately 10% lower with everolimus compared with MMF, with a notably lower rate of CMV reported as serious infections among everolimus-treated patients (1.6% vs. 18.0% of MMF-treated patients). A reduced incidence of CMV infection with everolimus compared with mycophenolic acid has been reported previously in kidney transplantation [12,15], although the between-group difference in CMV infection in the present study was greater than in previous trials. This may have resulted from more frequent CMV testing at Japanese centers than is standard elsewhere. Reports in preclinical studies state that mTOR inhibition may promote differentiation of antiviral memory CD8 T cells [23,24], upregulate proinflammatory cytokines, downregulate anti-inflammatory cytokines, and boost major histocompatibility complex antigen presentation [25] effects that would be expected to contribute to reduced viral infection.

As a consequence of the low number of kidney transplants performed each year in Japan, the study size was small, with relatively low statistical power. However, the findings were remarkably similar to those observed in the large A2309 study, which used a similar design. As in A2309, the patients in this study were selected to be of relatively low immunological risk, and in addition were almost exclusively recipients of a living-donor graft. The results may therefore not be generalizable to a wider population. The core study was 12 months in duration, which may not have been adequate to fully examine the effect of an everolimus-based regimen on renal function. An extension phase will provide data to 24 months. One should also note that although an open-label design was mandatory because of the need to adjust drug doses based on trough concentrations in each patient, this does introduce the risk of reporting bias, particularly for AEs.

In conclusion, as compared with other countries, currently there are limited immunosuppressant options available for RTx patients in Japan. This study in *de novo* Japanese RTx patients demonstrated that everolimus (targeting a C0 of 3 to 8 ng/ml) with minimized cyclosporine exposure was non-inferior to MMF with standard-exposure cyclosporine in preventing efficacy failure to 12 months post transplant. Renal function did not differ significantly between the two groups, but was numerically higher in the everolimus cohort throughout the study. No safety concerns specific to Japanese patients were observed. While relatively small, this trial benefits from a multicenter, randomized design and is the first to validate the results of the large A2309 study in a Japanese population. The findings indicate that cyclosporine minimization facilitated by everolimus is a viable immunosuppressive regimen for Japanese recipients of a kidney transplant and may also contribute to the long-term maintenance of good graft function and patient survival.

## Abbreviations

AE: adverse event; BPAR: biopsy-proven acute rejection; C0: trough concentration; CI: confidence interval; CMV: cytomegalovirus; CNI: calcineurin inhibitors; ELISA: enzyme-linked immunosorbent assay; eGFR: estimated glomerular filtration rate; LTFU: loss to follow-up; MMF: mycophenolate mofetil; mTOR: mammalian target of rapamycin; RR: risk ratio; RTx: renal transplant.

## Competing interests

KT has links with the Novartis Speakers Bureau, and is an advisor. KU has links with Novartis Speakers Bureau. NY has links with Novartis Speakers Bureau. STa has links with Novartis Speakers Bureau. STe has links with Novartis Speakers Bureau. RT is a Novartis employee. CC-A is a Novartis employee. EK has links with Novartis Speakers Bureau for grant/research support and as an advisor, and a special advisor for Otsuka Pharmaceutical Factory Inc. (Naruto, Japan) from 2009.

## Authors’ contributions

KT, KU, STa, NY, STe, and EK participated in the research design and performance of the research. RT and CC-A performed the data analysis. All authors read and approved the final manuscript.

## Supplementary Material

Additional file 1 Figure showing a Kaplan–Meier plot for the proportion of patients free from composite efficacy failure to cut-off date (intent-to-treat population – 12-month analysis). Click here for file

Additional file 2 **Figure showing the eGFR (Modification of Diet in Renal Disease (MDRD)) over time by treatment group (intent-to-treat population).** At each visit box-plots are displayed, with the means joined by a horizontal line. The whiskers are shown at the 10th and 90th percentiles. EMV, everolimus. Click here for file

Additional file 3 Table presenting a summary of infections over 12 months of treatment (safety population). Click here for file

Additional file 4 Table presenting incidence rates of patients with CMV infections by donor/recipient CMV status at baseline and CMV prophylaxis (safety population). Click here for file
