# Supplementary material for: Efficacy and safety of concentration-controlled everolimus with reduced-dose cyclosporine in Japanese de novo renal transplant patients: 12-month results
Source: Transplant Res. 2013 Jul 16;2:14. doi: 10.1186/2047-1440-2-14 (PMC3718642; doi:10.1186/2047-1440-2-14)
Supplement: Additional file 3 — Table presenting a summary of infections over 12 months of treatment (safety population). [file 2047-1440-2-14-S3.doc]

**Supplementary Table 1: Summary of infections over 12 months of treatment (safety population)**

| **Type of infection** | **Everolimus**  **N=61**  **n (%)** | **MMF**  **N=61**  **n (%)** |
| --- | --- | --- |
| Overall infections | 50 (82.0) | 57 (93.4) |
| Viral infection | 17 (27.9) | 49 (80.3) |
| Cytomegalovirus, NOS | 9 (14.8) | 42 (68.9) |
| Human herpes simplex virus, NOS | 4 (6.6) | 6 (9.8) |
| BK virus | 2 (3.3) | 2 (3.3) |
| Influenza virus, NOS | 2 (3.3) | 1 (1.6) |
| Adenovirus, NOS | 1 (1.6) | 3 (4.9) |
| Human herpes virus 3 | 1 (1.6) | 3 (4.9) |
| Human herpes virus 4 | 1 (1.6) | 0 (0.0) |
| Influenza virus A | 0 (0.0) | 1 (1.6) |
| JC virus | 0 (0.0) | 1 (1.6) |
| Varicella virus, NOS | 0 (0.0) | 1 (1.6) |

MMF, mycophenolate mofetil; NOS, not otherwise specified
