# Supplementary material for: Efficacy and safety of concentration-controlled everolimus with reduced-dose cyclosporine in Japanese de novo renal transplant patients: 12-month results
Source: Transplant Res. 2013 Jul 16;2:14. doi: 10.1186/2047-1440-2-14 (PMC3718642; doi:10.1186/2047-1440-2-14)
Supplement: Additional file 4 — Table presenting incidence rates of patients with CMV infections by donor/recipient CMV status at baseline and CMV prophylaxis (safety population). [file 2047-1440-2-14-S4.doc]

**Supplementary Table 2: Incidence rates of patients with CMV infections by donor/recipient CMV status at baseline and CMV prophylaxis (Safety population)**

| **D/R CMV status** | **CMV prophylaxis** | **EVR 1.5 mg (N=61)**  **n/M (%)** | **MMF**  **(N=61)**  **n/M (%)** |
| --- | --- | --- | --- |
| Total | Yes  No | 1/1 (100.0)  8/60 (13.3) | 6/6 (100.0)  36/55 (65.5) |
| D+/R+ | Yes  No | 0/0 (0.0)  2/39 (5.1) | 3/3 (100.0)  25/39 (64.1) |
| D+/R- | Yes  No | 1/1 (100.0)  5/7 (71.4) | 3/3 (100.0)  6/8 (75.0) |
| D-/R+ | No | 1/8 (12.5) | 1/1 (100.0) |
| D-/R- | No | 0/5 (0.0) | 1/3 (33.3) |
| Other | No | 0/1 (0.0) | 3/4 (75.0) |

CMV, cytomegalovirus; D, donor; EVR, everolimus; MMF, mycophenolate mofetil;

R, recipient
